# Supplementary material for: A 3D Tissue Model of Traumatic Brain Injury with Excitotoxicity That Is Inhibited by Chronic Exposure to Gabapentinoids
Source: Biomolecules. 2020 Aug 17;10(8):1196. doi: 10.3390/biom10081196 (PMC7463727; doi:10.3390/biom10081196)
Supplement: Supplementary file 1 [file biomolecules-10-01196-s001.pdf]

Supplemental Figures and Captions:

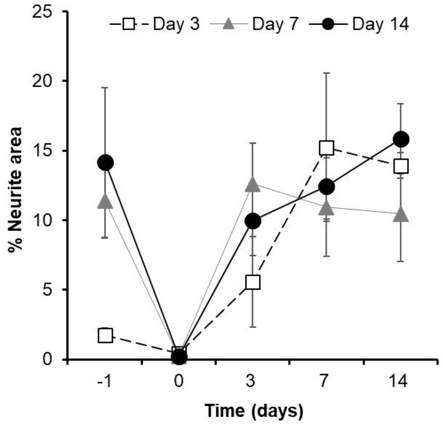

Figure 1. Percentage neurite area within the central window over time as a function of day of 2mm (mild) laceration. Whether lacerated 3 (squares), 7 (triangles), or 14 (circles) days post-seeding, neurite area within the central window was re-established to baseline levels within 3 days of injury. It was concluded that the 2mm injury did not induce a long-lasting pattern of damage. Means and standard error of the means are displayed.

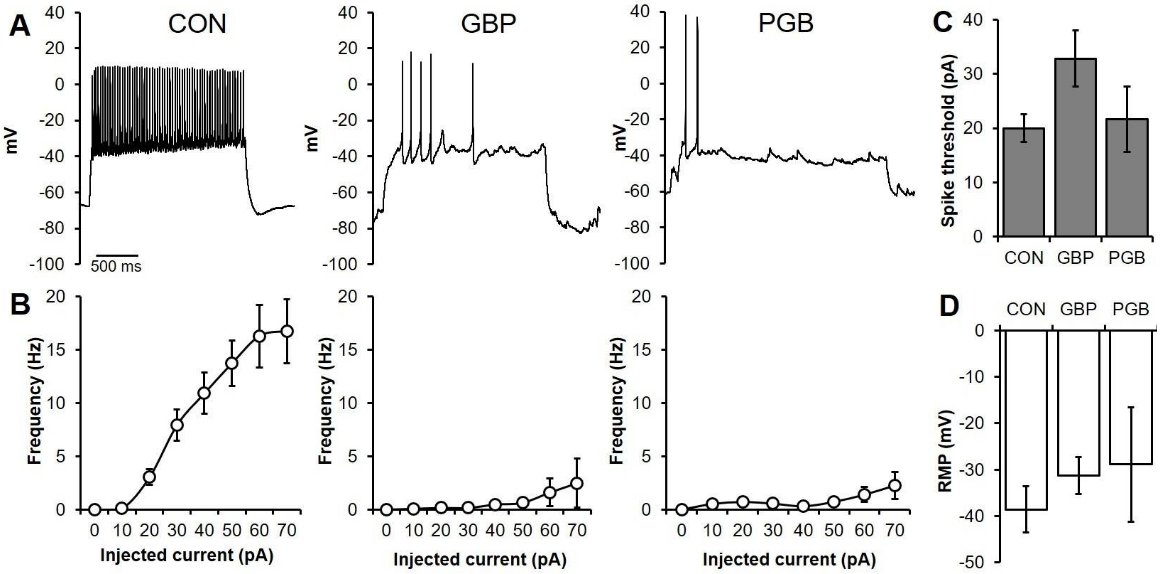

Figure 2. Electrophysiological characterization of acute effects of GBP and PGB on primary cortical neurons. (A) Representative traces of action potential recordings in the controls (CON, left), 100μM GBP-exposed (middle), and 100μM PGB-exposed (right) groups upon injection of +50 pA of current. All recordings were made after clamping the potential at -70 mV. (B) Frequency of spikes were quantified as a function of the magnitude of the injected current (pA) for each condition (n=6/group); means and SEMs are reported. (C)Spike thresholds and (D) resting membrane potentials (RMP) are also reported for each condition (means and SEMs).

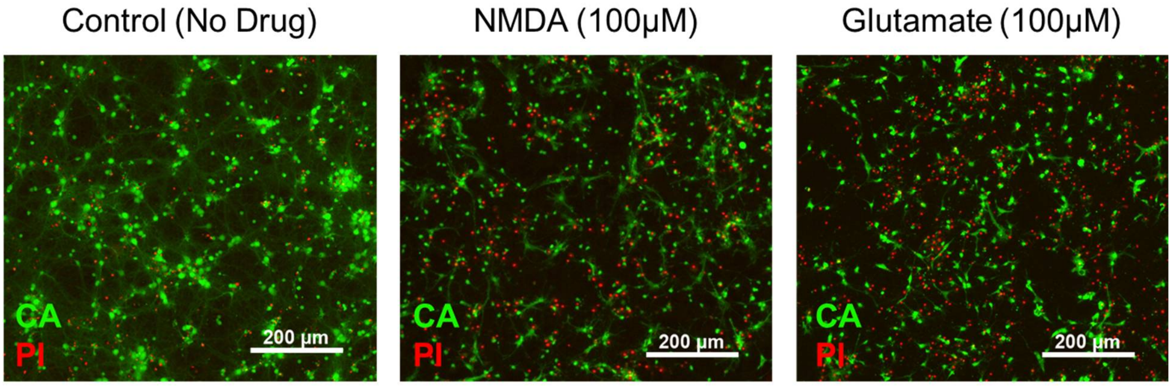

Figure 3. Live-dead fluorescent imaging of cells exposed to excitotoxic compounds. Examples of CA- and PI-positive cell populations within control (no drug; left), 100 μM NMDA (middle), and 100 μM glutamate (right) exposure conditions, demonstrating excitotoxicity. Scale bars represent 200μm increments.

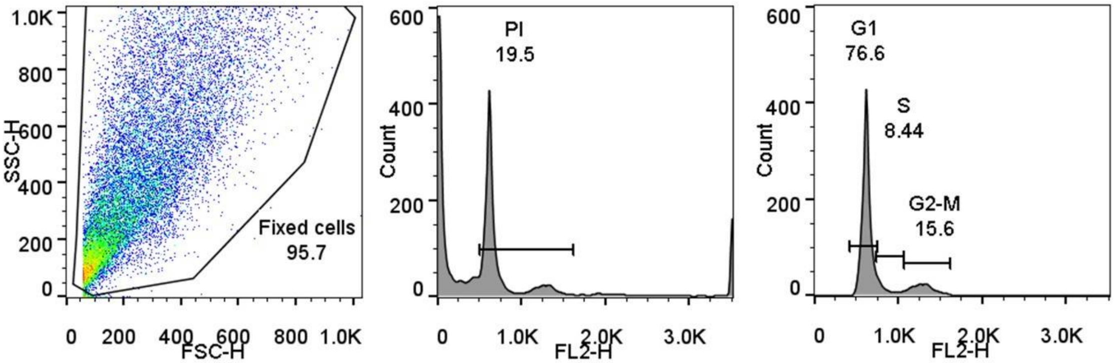

Figure 4. Gating strategy for PI analysis. Fixed cells were first gated by size (FSC) and granularity (SSC) (left). This population was then further filtered for debris through manual selection of the two gaussian-like peaks characteristic of the PI stain, resulting in ~20% of events that were intact cells (center). Lastly, the percentages of cells in various phases of the cell cycle were quantified through a manual definition of the G1, S, and G2-M phases (right).
